# Body Mass Index and Serum Proteomic Profile in Breast Cancer and Healthy Women: A Prospective Study

**DOI:** 10.1371/journal.pone.0049631

**Published:** 2012-11-30

**Authors:** Vito Michele Garrisi, Antonio Tufaro, Paolo Trerotoli, Italia Bongarzone, Michele Quaranta, Vincenzo Ventrella, Stefania Tommasi, Gianluigi Giannelli, Angelo Paradiso

**Affiliations:** 1 Clinical Experimental Oncology Department, National Cancer Research Centre, Istituto Tumori “Giovanni Paolo II”, Bari, Italy; 2 Biomedical Science and Human Oncology Department, University of Bari Medical School, Bari, Italy; 3 Experimental Oncology and Laboratory Department, IRCCS Foundation Istituto Nazionale Tumori, Milano, Italy; 4 Women's Department, National Cancer Research Centre, Istituto Tumori “Giovanni Paolo II”, Bari, Italy; 5 Department of Emergency and Organ Transplantation, Section of Internal Medicine Allergology and Immunology, University of Bari Medical School, Bari, Italy; 6 Scientific Directorate, National Cancer Research Centre, Istituto Tumori “Giovanni Paolo II”, Bari, Italy; University of Bari & Consorzio Mario Negri Sud, Italy

## Abstract

Epidemiological studies suggest a possible association between BMI, diagnosis and clinical-pathological breast cancer characteristics but biological bases for this relationship still remain to be ascertained. Several biological mechanisms play a role in the genesis and progression of breast cancer. This study aimed to investigate relationships between BMI and breast cancer diagnosis/progression in a Southern Italian population and to try to interpret results according to the serum proteomic profile of healthy and breast cancer patients.

BMI, presence or absence of breast cancer and its clinical-pathological characteristics were analyzed in a series of 300 breast cancer women and compared with those of 300 healthy women prospectively. To investigate whether obesity is associated with alterations in serum protein profile, SELDI-ToF approach was applied.

Alcohol consumption (22.7% vs 11.3%; p<0.001) and postmenopausal status (65.7% vs 52%; p<0.001) but not BMI resulted significantly different in patients vs controls. Conversely, BMI was significantly associated with a larger-tumour size (BMI> = 30 respect to normal weight: OR = 2.49, 95% CI 1.25–4.99, p = 0.0098) and a higher probability of having positive axillary lymph node (OR = 3.67, CI 95% 2.16–6.23, p<0.0001). Multivariate analysis confirmed the association of breast cancer diagnosis with alcohol consumption (OR = 2.28;CI 1.36–3.83; p<0.0018). Serum protein profile revealed the presence of significant (p-value <0,01) differentially expressed peaks m/z 6934, m/z 5066 in high BMI breast cancer patients vs healthy subjects and m/z 6934, m/z 3346 in high vs low BMI breast cancer patients.

The analysis of pathological features of cancer indicates that normal weight women have a significantly higher probability of having a smaller breast cancer at time of diagnosis and negative axillary lymph nodes while increased BMI is associated with an altered protein profile in breast cancer patients. Further studies to identify specific proteins found in the serum and their role in breast cancerogenesis and progression are in progress.

## Introduction

In the last recent years, body-weight has been suggested as a possible factor associated with breast cancer [1] and, nowadays, the link between obesity and postmenopausal breast cancer can be confirmed. This link has generally been attributed to the action of some hormones relevant in fat metabolism and in particular to direct serum concentration increase of bioavailable oestradiol [2]. In a preliminary study conducted in a small group of women from south Italy, we confirmed this hypothesis and the relationship between obesity and breast cancer [3]. Several biological mechanisms, variously implicating hormone regulation of breast cell growth and related to weight and mass of the body, may play a role in the genesis and progression of breast cancer. In particular, the concentration of circulating estrogens, which is related to both increased adipose tissue mass and up-regulation of aromatase enzyme, has been reported to be a contributing factor towards the increased risk for hormone receptor – positive breast cancer in obese women [4]. Obesity is also associated with reduced plasma levels of sex hormone – binding globulin, a protein responsible for the biological activity of estrogens [5]. Notably, obesity is also a cause of insulin resistance, which is characterized by hyperinsulinemia [6] that, in patients with breast cancer, has been associated with an higher probability of distant recurrence and increased mortality [7]. The mitogenic, antiapoptotic, and proangiogenic properties of insulin have been suggested as an explanation of its implication in cancer progression; furthermore, insulin is able to stimulate the synthesis of IGF-1, which has multiple effects that have been linked to tumor growth and metastasis [8].

Moreover, several reports, including a recent large retrospective cohort study [4], have shown that being overweight worsens breast cancer outcome; in particular, obese women were reported to have greater disease morbidity, higher recurrence rate, increased contralateral breast cancer occurrence, wound complications after breast surgery, and lymphedema. Poorer outcome associated with breast cancer has been related to delayed disease detection [10], a more aggressive disease at diagnosis, and/or a higher likelihood of treatment failure [2]. Interestingly, these recently reported characteristics do not vary in ethnically diverse postmenopausal women [1]. Lastly, Ewertz [9] provides important evidence that adjuvant treatment could also be effective to differing degrees in obese and normal weight women.

In spite of the plethora of information concerning the possible biological mechanisms relating body weight/mass with breast cancer, much remains to be understood. In particular, due to the ubiquitary and relevant presence of fat in the body, serum has to be viewed as an important and unique source of information which has not been sufficiently explored. Moreover, despite advances in screening, diagnosis, and treatment of breast cancer, many challenges remain; no reliable serum biomarker has been discovered, which might be a result, in part, of the pathogenic heterogeneity of breast cancer [11].

SELDI ToF – MS is a high throughput technique based on the chromatographic separation of proteins according to their physical characteristics (i.e. hydrophobic, hydrophilic, acidic, basic, metal affinity) which allows analysis of serum proteomic profiles with high sensitivity and good reproducibility [12]. Many groups including ourselves have already utilized this approach to successfully analyze sera profiles from patients with solid tumours in order to predict diagnosis [13] and response to therapy [14].

The aim of the present study was: 1) to analyze Body Mass Index (BMI), as a risk factor for breast cancer and/or predictive factor for its pathological characteristics in a prospective consecutive and monoinstitutional series of 300 breast cancer women compared to 300 healthy women; 2) to perform serum proteomic profiles by SELDI-ToF-MS in the same consecutive and prospective series of subjects in order to discover serum protein specifically related with breast cancer and BMI.

## Materials and Methods

### Ethics Statement

The study was performed with the approval of the Ethics Committee of National Cancer Research Center “Istituto Tumori Giovanni Paolo II” of Bari, Italy. Each individual involved in the study signed an informed consent form authorizing the Institute to utilize their biological tissues for research purposes.

### Patients and Methods

All the subjects enrolled in the study were consecutively and prospectively observed between 2004 and 2006 in the outpatient Service of Senology-Radiodiagnostic Unit of the National Cancer Research Centre “Istituto Tumori Giovanni Paolo II”.

Subjects eligible for the study included 1) patients who had a positive mammographic diagnosis and, successively, a surgical histological diagnosis of breast cancer and 2) 300 healthy women who had, in the same period, a negative mammography for screening purposes confirmed by at least three previous mammographies in the preceding three years.

For each subject involved in the study the following information were collected: a) alcohol intake was classified as Yes or No Alcohol consumption (cut-off 50 ml/day); b) smoking habit classified as non smokers or smokers (cut-off 20 cigarettes a day) c) premenopausal status if having menstrual cycles in the last twelve month period preceding the date of the interview. d) cancer familiarity if at least one first-degree relative reported a breast/ovarian cancer.

After having measured weight and height, body mass index (BMI) was calculated by dividing body weight (expressed in kg) by the body surface. BMI threshold values suggested by the World Health Organization were adopted, with each subject being defined as normal (BMI<24.9, Low BMI), over weight (BMI 25><29.9, Medium BMI) or obese (BMI>30, High BMI).

Subjects having access to the Senology-Diagnostic Unit were interviewed before their Mammography, and, afterwards, a blood sample was taken and sent to the Experimental Laboratory to be aliquoted and frozen according to SOPs. Subjects with suspected or positive Mammography underwent to surgical diagnosis and, eventually, primary treatment in the Women's Department of the Institute; from the operating room, the removed breast tissue was sent to the Histopathology Unit for further histological analyses. Each sample was successively staged according to pTNM criteria of the AJCC [15]. Cytohistological grade was defined according to Fischer [16]. Hormone receptors were analysed by himmunohistochemical assay and cases were considered positive if more than 10% of cells resulted stained for ER or PgR. Proliferative activity of the breast cancer was analyzed by immunohistochemistry by analysis of the percentage of cells positive for MIB-1 as previously reported [17]. Ca 15.3 serum level analysis through immunoenzymatic assay was performed by the Pathology Service of the Institute. A cut-off of 40 u/ml was utilized to classify each sample as being at a normal or high Ca 15.3 level.

### Serum Protein Profiling

In 140 healthy subject and 138 breast cancer women serum protein profiling was performed using Surface Enhanced Laser Desorption Ionization – Time of Flight (so called SELDI ToF) Mass Spectrometry (MS) technology. SELDI TOF – MS is a high throughput technique based on the chromatographic separation of proteins according to their physical characteristics (i.e. hydrophobic, hydrophilic, acidic, basic, metal affinity). In our study, metal affinity IMAC 30 protein chip and cationic exchange CM 10 protein chip surfaces were used. In order to compare serum profile of high BMI breast cancer patients and High BMI normal subjects 42 and 37 serum samples respectively were analyzed. In order to compare the serum profile of breast cancer patients with high and low BMI, 42 serum samples from as many high BMI breast cancer patients and 58 with low BMI were analyzed.

The two protein chips types were prepared according to the manufacturer's instructions. All the chips were read by adopting the same protocol (laser energy 5000 nJ, matrix attenuation 500 Da, focus mass 11.000, sample rate 400, acquired mass range from 2500 to 20,000 Da). The software was externally calibrated using the all in one protein standard kit (Bio-Rad Laboratories, Inc. USA) specific for low molecular weight molecules and spectra were generated in the mass to charge range of 2500 to 20,000 Da. Automatic peak detection was performed along the entire spectrum using Protein Chip Data Manager program (version 4.1; Bio-Rad Laboratories, USA) with the following settings: signal/noise ratio (first pass), 3; minimum peak threshold, 10%; cluster mass window, 0.3%; signal/noise ratio (second pass), 1.5. Following peak detection and clustering, average peak intensities, were calculated for all. Peak expression differences between spectra were calculated by the Expression Difference Mapping (EDM), using the non-parametric Kruskall-Wallis test. This analysis allows to observe how many peaks are differentially expressed and, moreover, how many of them are statistically significant. The software calculated m/z feature and the intensity measure (expressed in μA which is cautiously considered a concentration measure). *P-*values<0.03 were considered statistically significant.

### Statistical Analysis

Quantitative variables were summarized as mean and standard deviation. Comparison between independent groups was performed by means of t-test, given Gaussian distribution of data. The difference in percentage between independent groups and the relationship between variables were analyzed by means of Pearson's Chi-square test.

Multivariate logistic regression models were performed to evaluate risk for breast cancer with BMI, alcohol consumption, age, cancer familiarity, smoking habit, menopausal state included in the model.

A further multivariate logistic model was preformed to evaluate the risk of having a larger tumor size; independent variables included in the analysis were BMI, alcohol consumption, age, cancer familiarity, smoking habit, menopausal status, axillary lymph node status, and Ca15.3 level.

The statistical analysis was performed using SPSS software (SPSS inc. Italy) version 9 and SAS 9.2.

## Results

### BMI and breast cancer diagnosis

The clinical features of the cohort of patients affected by breast cancer and of the healthy women (control group) are reported in Table 1. The two groups did not show any significant difference in frequency of different BMI categories even though a trend for higher percentage of high BMI cases in breast cancer women was clearly evident (p = 0.0721 by chisquare test). On the contrary, we showed a significantly higher percentage of breast cancer patients consuming alcohol routinely (22.7% vs 11.3%, p = 0.0002) as well as higher percentage of women in postmenopausal status (65.7% vs 52% p = 0.0002) with respect to control subjects.

**Table 1:** Clinicopathological features in a case study of 300 patients affected by breast cancer and 300 control with negative mammography result.

Feature		Breast Cancer n = 300	Control n = 300	*P*-value
BMI	Low	111 (37,0%)	129 (43,0%)	0,0721*
	Medium	115 (38,3%)	119 (39,7%)	
	High	74 (24,7%)	52 (17,3%)	
Family History	yes	188 (62,7%)	199 (66,3%)	0,348*
	no	112 (37,3%)	101 (33,7%)	
Alcohol consumption	yes	68 (22,7%)	34 (11,3%)	0,0002*
	no	232 (77,3%)	266 (88,7%)	
Smoking habit	yes	66 (22,0%)	85 (28,3%)	0,074*
	no	234 (78,0%)	215 (71,7%)	
Menopausal status	Pre	103 (34,3%)	144 (48,0%)	0,0007*
	Post	197 (65,7%)	156 (52,0%)	
Average age (years)		58,1	52,2	

* : χ^2^ test.

In Table 2, the results of a further analysis of the relationship between BMI and breast cancer characteristics, separately for pre and postmenopausal subgroups, are reported; while in premenopausal women no significant differences were evident, we observed a trend in terms of association between high BMI value and breast cancer presence.

**Table 2:** BMI and pausal status in 300 subjects affected by breast cancer and 300 control cases.

Pausal Status		Breast cancer patients n = 300	Control cases n = 300	*P*-value
B.M.I. premenopause				0.915
	Low	55 (53,4%)	73 (50,7%)	
	Medium	35 (33,0%)	52 (36,1%)	
	High	13 (12,6%)	19 (13,2%)	
B.M.I. postmenopause				0.091
	Low	56 (28,4%)	56 (35,9%)	
	Medium	80 (40,6%)	67 (42,9%)	
	High	61 (30,0%)	33 (21,2%)	

Mantel – Haenszel χ^2^ p<0.05.

In order to check for factors independently related to the presence of breast cancer, a multivariate logistic model was further performed (Table 3). Only alcohol consumption (more than 50 ml/day) resulted independently associated with a significantly higher probability having cancer (OR = 2.28; CI 1.36–3.83; p<0.001).

**Table 3:** Logistic multivariate model with breast cancer as dependent variable.

Variables	Category	Odds Ratio	Confidence Interval 95%	*P*-value
Age	41–50 vs < = 40	0.67	0.35–1.27	0.2203
	> = 51 vs < = 40	0.9	0.41–1.97	0.7983
Smoking habit	yes vs no	0.82	0.55–1.21	0.3165
Alcohol consumption	yes vs no	2.28	1.36–3.83	0.0018
Family history	yes vs no	0.9	0.64–1.28	0.5656
Menopausal status	Post vs pre	1.33	0.77–2.29	0.3089
BMI	High vs Low	1.44	0.91–2.27	0.121

### BMI and clinical-pathological characteristics of breast cancer

When BMI was analyzed with respect to the clinical-pathological characteristics of breast cancer (Table 4), we demonstrated, again, that an higher BMI was significantly associated with a post-menopausal status (p<0.0001), with a larger tumor diameter (p<0.0013), with an higher serum level of Ca15.3 ((p<0.0002) and with presence of positive metastatic axillary lymph nodes (p<0.003).

**Table 4:** Clinical-pathological breast cancer characteristics and BMI, χ^2^ test was used to calculate p-value.

Features		Tot (%)	Low (n = 111)	Medium (n = 115)	High (n = 74)	*P*-value
Menopausal status	Pre	34	55 (49,5%)	35 (30,4%)	13 (17,6%)	<0.0001
	Post	66	56 (50,5%)	80 (69,6%)	61 (82,4%)	
Tumor size	≤2 cm	43	60 (56,6%)	39 (36,1%)	23 (32,4%)	0.0013
	>2 cm	57	46 (43,4%)	69 (63,9%)	48 (67,6%)	
Cytohistological grading	1	9	14 (13.5%)	8 (7.4%)	2 (3.1%)	0.0631
	2	42	43 (41.3%)	51 (47.2%)	23 (35.9%)	
	3	49	47 (45.2%)	49 (45.4%)	39 (60.0%)	
Marker Ca15.3 (U/ml)	>40	21	15 (14.3%)	18 (16.1%)	27 (38.1%)	0.0002
	< = 40	79	90 (85.7%)	94 (83.9%)	44 (61.9%)	
Immunohistochemical markers	ER +	75	79 (73,2%)	88 (80,7%)	51 (70,8%)	0.249
	ER −	25	29 (26.8%)	21 (19.3%)	21 (29.2%)	
	PgR +	60	61 (55.9%)	70 (64.2%)	43 (59.7%)	0.4604
	PgR −	40	48 (44.1%)	39 (35.8%)	29 (40.3%)	
	Mib +	50	59 (54.1%)	48 (44.1%)	38 (52.8%)	0.2843
	Mib −	50	50 (45.9%)	61 (55.9%)	34 (47.2%)	
Axillary	Positive	55	43 (40,6%)	63 (62,4%)	38 (61,3%)	0.003
	Negative	45	63 (59,4%)	38 (37,6%)	24 (38.7%)	

At a further logistic analysis (data not shown), the only factors significantly associated with a larger tumor size (T>2 cm) were high BMI (OR = 2.49, 95% CI 1.25–4.99, p = 0.009), and a positive axillary nodal status (OR = 3.67, CI 95% 2.16–6.23, p<0.0001). Even more interestingly, high BMI resulted independently associated with higher tumor biomarker Ca15.3 level (data not shown).

### BMI and serum proteomic profile

Based on previous results confirming the relationship between BMI and breast cancer diagnosis and outcome, with the SELDI-TOF approach we further explored which serum factor, if any, could be found associated with breast cancer. To identify serum protein associated with breast cancer and to identify serum proteins related with different clinical outcome, we then performed the following comparisons: a) serum profiling from high BMI subjects with or without breast cancer; b) serum profiling from breast cancer women with low or high BMI.

Results of SELDI-TOF analysis of serum from high BMI subjects with or without breast cancer are reported in Table 5. Sixty six peaks from IMAC 30 and 64 from CM 10 protein chip resulted differentially expressed, eight of which statistically significant. Even more importantly, all peaks considered, are under expressed in cancer with respect to the control; the most significant peak by the metallic affinity chip was at 6934 m/z,given the highly different average peak intensity (1,621 vs 2,086; p<10-5). The particularly high expression of the proteins characterized by peaks m/z 5065 (11.1825 vs 14.383 respectively in high BMI breast cancer patients vs high BMI healthy subjects) and at m/z 5338 are particularly noteworthy. Similarly, by CM10 chip recognizing proteins through cationic exchange, the most significant peak was at 5066 m/z with an average peak intensity different at 10^−4^ (2.21 vs 3.884).

**Table 5:** Comparison of peak intensities average (μA) between high BMI breast cancer patients and healthy subjects.

Peaks m/z	Breast Cancer Patients (n = 42)	Healthy Subjects (n = 37)	*P*-value
6934*	1,621	2,086	0,000492
5065*	11,1825	14,383	0,00128
5066**	2,21	3,884	0,00011
4644**	10,07135	14,23982	0,0009436
5338**	13,01519	17,74265	0,00389
16678**	11,895	14,504	0,00459
10262**	1,175	1,85	0,007
4283**	6,63	8,585	0,00824

Only significant peaks (P-value<0.01) are reported.

**Legend:**

* = From IMAC 30 Dataset.

** = From CM 10 Dataset.

The comparison between breast cancer patients with high BMI and low BMI is reported in Table 6; in this case IMAC 30 highlighted 55 differentially expressed, while CM 10 61. Four peaks were statistically significant. The most significant different peak expressed was at 6934 m/z (intensity peak average 1,621 vs 2,235; p<10^−5^). Once again, data in the Table show that the majority of peaks had low expression in high BMI patients.

**Table 6:** Comparison of peak intensities average (μA) between Breast cancer patients with high BMI and low BMI.

Peaks m/z	High BMI (n = 42)	Low BMI (n = 58)	*P*-value
6934*	1,621	2,235	0,000045723
11733*	7,35	5,66	0,000842
2792*	1,723	3,056	0,00399
3346**	2,391	3,86007	0,000010954

Only significant peaks (P-value<0.01) are reported.

**Legend:**

* = From IMAC 30 Dataset.

** = From CM 10 Dataset.

## Discussion

The association between BMI and breast cancer risk development as well with pathological features of more aggressive disease is well known. However, the biological basis for this role is far from being definitively clarified. To our knowledge, despite the amount of well characterized informations about the influence of hormones and, life style on breast cancer onset, the link between serum proteomic profile, BMI, and breast cancer has not yet been greatly investigated. In this study we show for the first time the role of BMI in breast cancer in a case series of women with specific geographical origins. The main findings of our study are 1) BMI was statistically different in breast cancer patients compared to controls; 2) BMI was significantly associated with a higher risk of developing a more invasive and aggressive disease; patients with higher BMI are also those expressing a protein profile peaks characterized by different m/z values but the related proteins remain to be identified. The main weakness of our study is the lack of identified protein peaks. We realized the importance of this step, however, and this issue is currently under further investigation and will likely be described in future reports.

As shown in Table 5, such differences were sharper comparing high BMI breast cancer patients and healthy subjects. Conversely, when high and low BMI breast cancer patients were compared, the differences were slight. This evidence seems to suggest that the main differences are due to the presence of tumor and not to obesity-related proteins. Therefore, according to this assumption we suggest that BMI might not influence the tumor onset trough the production of specific proteins that can be detected in serum.

The most significant differentially expressed peak was at 6934 m/z which resulted under-expressed with a lower value in high BMI and breast cancer patients, (see Table 5 and 6). To our knowledge the identity of this peak, is unknown, and therefore identification is required in order to clarify its characteristics. In addition, both IMAC30 and CM10 recognized the same peak (at m/z 5065 and 5066 respectively) with different intensity levels (probably due to difference of the chip surface); at this time we do not know which protein is referred to this peak but identification analysis is now in progress. We hypothesize that serum proteins referring to these peaks could be responsible for the possible cancerogenic role that obesity plays at least in some subgroups of breast cancer women. As further consideration, we can anticipate that in another analysis of different group of patients, the peak at 11730 m/z and the peak at 5066 m/z (data not shown) were confirmed to be differentially expressed between cancer and healthy subjects, thus suggesting the association with cancer. This interesting evidence, is worthy of note and will be the subject of a future project thus focusing on peak characterization. Our preliminary analysis, confirmed the possibility of this characterization.

Another major strength of this study is the fact that we have analyzed the role of BMI as a cancerogenesis factor in cancer diagnosis and disease progression. We know that body weight has already been proved to be related to voluptuary habits (smoking, alcohol consumption, diet), and socio cultural factors (marital, educational and economic status, ethnic origin, physical exercise) [18]. The results of a study carried out on women in North Italy suggested that vegetable and olive oil consumption has a protective role against breast cancer in a Mediterranean population [19]. In our previous study carried out in a female population from the same area than the present series, a significant association between obesity and breast cancer risk in postmenopausal women regardless of diabetes mellitus was reported [4]. On the contrary, in a study considering African American women an association was found between body mass index and breast cancer risk independently from pausal status [20]. In conclusion, while other papers emphasized the association between high BMI and breast cancer risk, we further hypothesize that BMI could have a direct role in tumor growth stimulation.

## Conclusions

The analysis of pathological features of cancer indicates that normal weight women have a significantly higher probability of having a smaller breast cancer at time of diagnosis and negative axillary lymph nodes. Increased BMI is associated with an altered protein profile in breast cancer patients. Further studies are therefore required to identify these proteins and to assess their biological role in body weight and breast cancer natural history.
